# Quantitative-Voronovskaya and Grüss-Voronovskaya type theorems for Szász-Durrmeyer type operators blended with multiple Appell polynomials

**DOI:** 10.1186/s13660-017-1520-y

**Published:** 2017-10-03

**Authors:** Trapti Neer, Purshottam Narain Agrawal

**Affiliations:** Department of Mathematics, Indian Institute of Technology Roorkee, Roorkee, 247667 India

**Keywords:** 41A25, 41A36, Steklov mean, first and second order modulus of continuity, weighted modulus of continuity, Grüss-Voronovskaya type theorem, functions of bounded variation

## Abstract

In this paper, we establish a link between the Szász-Durrmeyer type operators and multiple Appell polynomials. We study a quantitative-Voronovskaya type theorem in terms of weighted modulus of smoothness using sixth order central moment and Grüss-Voronovskaya type theorem. We also establish a local approximation theorem by means of the Steklov means in terms of the first and the second order modulus of continuity and Voronovskaya type asymtotic theorem. Further, we discuss the degree of approximation by means of the weighted spaces. Lastly, we find the rate of approximation of functions having a derivative of bounded variation.

## Introduction

For $f\in C(R_{0}^{+})$ and $x\in R_{0}^{+}$ ($R_{0}^{+}=[0,\infty)$), Szász [1] introduced the well-known operators

1 $$\begin{aligned} S_{n}(f;x)=e^{-nx}\sum _{k=0}^{\infty}\frac{(nx)^{k}}{k!}f\biggl(\frac{k}{n} \biggr), \end{aligned}$$

such that $S_{n}( \vert f \vert ;x)< \infty$. Several generalizations of Szász operators have been introduced in the literature and authors have studied their approximation properties. In [2], the author considered Baskakov-Szász type operators and studied the rate of convergence for absolutely continuous functions having a derivative equivalent with a function of bounded variation. In [3], the authors introduced the *q*-Baskakov-Durrmeyer type operators and studied the rate of convergence and the weighted approximation properties. In [4] the authors proposed the *β*-operators based on *q*-integers and established some direct theorems by means of modulus of continuity and also studied the weighted approximation and better approximation using King type approach. For exhaustive literature on approximation by linear positive operators one can refer to [5–7] and the references therein.

Now let us recall some results on multiple Appell polynomials [8]. Let $g(z)=\sum_{n=0}^{\infty}a_{n}z^{n}$, $g(1)\ne1$, be an analytic function in the disc $\vert z \vert \leq r$, $r>1$ and $p_{k}(x)$ be the Appell polynomials having the generating function $g(u)e^{ux}=\sum_{k=0}^{\infty}p_{k}( x)u^{k}$, with $g(1)\neq0$ and $p_{k}(x)\geq0$, $\forall x\in R_{0}^{+}$.

Jakimovski and Leviatan [9] proposed a generalization of Szász-Mirakjan operators by means of the Appell polynomials as follows:

2 $$\begin{aligned} P_{n}(f;x)=\frac{e^{-nx}}{g(1)}\sum _{k=0}^{\infty}p_{k}(n x)f \biggl( \frac {k}{n} \biggr). \end{aligned}$$

For $g(u)=1$, these operators reduce to Szász-Mirakjan operators (1).

A set of polynomials $\{p_{k_{1},k_{2}}(x)\}_{k_{1},k_{2}=0}^{\infty}$ with degree $k_{1}+k_{2}$ for $k_{1},k_{2}\geq0$ is called multiple polynomial system (multiple PS) and a multiple PS is called multiple Appell if it is generated by the relation

3 $$\begin{aligned} A(t_{1},t_{2})e^{x(t_{1}+t_{2})}= \sum _{k_{1}=0}^{\infty}\sum_{k_{2}=0}^{\infty}\frac {p_{k_{1},k_{2}}(x)}{k_{1}!k_{2}!}t_{1}^{k_{1}}t_{2}^{k_{2}}, \end{aligned}$$

where *A* is given by

4 $$\begin{aligned} A(t_{1},t_{2})= \sum _{k_{1}=0}^{\infty}\sum_{k_{2}=0}^{\infty}\frac {a_{k_{1},k_{2}}}{k_{1}!k_{2}!}t_{1}^{k_{1}}t_{2}^{k_{2}}, \end{aligned}$$

with $A(0,0)=a_{0,0}\neq0$.

### Theorem 1.1

*For multiple PS*, $\{p_{k_{1},k_{2}}(x)\}_{k_{1},k_{2}=0}^{\infty}$, *the following statements are equivalent*:

- $\{p_{k_{1},k_{2}}(x)\}_{k_{1},k_{2}=0}^{\infty}$ *is a set of multiple Appell polynomials*.
- *There exists a sequence* $\{a_{k_{1},k_{2}}\}_{k_{1},k_{2}=0}^{\infty}$ *with* $a_{0,0}\neq0$ *such that*
  $$p_{k_{1},k_{2}}(x)= \sum_{r_{1}=0}^{\infty}\sum _{r_{2}=0}^{\infty}{k_{1}\choose r_{1}} {k_{2}\choose r_{2}}a_{k_{1}-r_{1},k_{2}-r_{2}}x^{r_{1}+r_{2}}. $$
- *For every* $k_{1}+k_{2}\geq1$, *we have*
  $$p'_{k_{1},k_{2}}(x)=k_{1}p_{k_{1}-1,k_{2}}(x)+k_{2}p_{k_{1},k_{2}-1}(x). $$

Varma [10] defined a sequence of linear positive operators for any $f\in C(R_{0}^{+})$, by

5 $$\begin{aligned} K_{n}(f;x)=\frac{e^{-nx}}{A(1,1)}\sum _{k_{1}=0}^{\infty}\sum_{k_{2}=0}^{\infty} \frac{p_{k_{1},k_{2}}(\frac{n x}{2})}{{k_{1}}!{k_{2}}!}f\biggl(\frac{k_{1}+k_{2}}{n}\biggr), \end{aligned}$$

provided $A(1,1)\neq0$, $\frac{a_{k_{1},k_{2}}}{A(1,1)}\geq0$ for $k_{1},k_{2} \in\mathbb{N}$, and the series (3) and(4) converge for $\vert t_{1} \vert < R_{1}$, $\vert t_{2} \vert < R_{2}$ ($R_{1}, R_{2}>1$), respectively.

For $\alpha>0$, $\rho>0$ and $f:R_{0}^{+}\rightarrow\mathbb{R}$, being integrable function, Pǎltǎnea [11] defined a modification of the Szász operators by

6 $$\begin{aligned} L_{\alpha}^{\rho}(f;x)=\sum _{k=1}^{\infty}s_{\alpha,k}(x) \int_{0}^{\infty}\frac {\alpha\rho e^{-\alpha\rho t}(\alpha\rho t)^{(k)\rho-1}}{\Gamma{(k)\rho}}f(t)\,dt+e^{-\alpha x} f(0),\quad x\in R_{0}^{+}. \end{aligned}$$

Motivated by [11], for $f\in C_{E}(R_{0}^{+})$, the space of all continuous functions satisfying $\vert f(t) \vert \leq K e^{a t}$ ($t\geq0$) for some positive constant *K* and *a*, we propose an approximation method by linking the operators (6) and the multiple Appell polynomials by

$$\begin{aligned} \mathcal{L}_{n}^{\rho}(f;x) =&\frac{e^{-nx}}{A(1,1)}\mathop{{\sum _{k_{1}}}\sum_{k_{2}}}_{k_{1}+k_{2}\geq1} \frac{p_{k_{1},k_{2}}(\frac{n x}{2})}{{k_{1}}!{k_{2}}!} \int_{0}^{\infty}\frac{n\rho e^{-n\rho t}(n\rho t)^{(k_{1}+k_{2})\rho-1}}{\Gamma{(k_{1}+k_{2})\rho}}f(t)\,dt \\ &{}+\frac{e^{-nx}}{A(1,1)}p_{0,0}\biggl(\frac{n x}{2}\biggr)f(0), \end{aligned}$$

and establish a quantitative Voronovskaya type theorem, a Grüss Voronovskaya type theorem, a local approximation theorem by means of the Steklov mean, a Voronovskaya type asymptotic theorem and error estimates for several weighted spaces. Lastly, we study the rate of convergence of functions having a derivative of bounded variation.

## Basic results

In order to prove the main results of the paper, we shall need the following auxiliary results.

### Lemma 2.1

*For* $K_{n}(t^{i};x)$, $i=0, 1, 2, 3, 4$, *we have*

$$\begin{aligned} \mathrm{(i)}\quad& K_{n}(1;x)=1, \\ \mathrm{(ii)}\quad& K_{n}(t;x)=x+\frac {A_{t_{1}}(1,1)+A_{t_{2}}(1,1)}{nA(1,1)}, \\ \mathrm{(iii)}\quad& K_{n}\bigl(t^{2};x\bigr)=x^{2}+ \frac{x}{n}\biggl\{ 1+\frac {2}{A(1,1)}\bigl(A_{t_{1}}(1,1)+A_{t_{2}}(1,1) \bigr)\biggr\} \\ &\hphantom{K_{n}\bigl(t^{2};x\bigr)=}{}+\frac{1}{n^{2}A(1,1)}\bigl\{ A_{t_{1}}(1,1)+A_{t_{2}}(1,1)+A_{t_{1}t_{1}}(1,1)+2A_{t_{1}t_{2}}(1,1)+A_{t_{2}t_{2}}(1,1) \bigr\} , \\ \mathrm{(iv)}\quad& K_{n}\bigl(t^{3};x\bigr)= x^{3}+\frac{3x^{2}}{n}\biggl\{ 1+\frac {1}{A(1,1)} \bigl(A_{t_{1}}(1,1)+A_{t_{2}}(1,1)\bigr)\biggr\} + \frac{x}{n^{2}}\biggl\{ 1 \\ &\hphantom{K_{n}\bigl(t^{3};x\bigr)=}{}+\frac {3}{A(1,1)}\bigl(2A_{t_{1}}(1,1)+2A_{t_{2}}(1,1)+A_{t_{1}t_{1}}(1,1)+2A_{t_{1}t_{2}}(1,1)+A_{t_{2}t_{2}}(1,1) \bigr)\biggr\} \\ &\hphantom{K_{n}\bigl(t^{3};x\bigr)=}{} +\frac{1}{n^{3}A(1,1)}\bigl\{ A_{t_{1}}(1,1)+A_{t_{2}}(1,1)+3A_{t_{1}t_{1}}(1,1)+6A_{t_{1}t_{2}}(1,1)\\ &\hphantom{K_{n}\bigl(t^{3};x\bigr)=}{}+3A_{t_{2}t_{2}}(1,1)+A_{t_{1}t_{1}t_{1}}(1,1)+A_{t_{2}t_{2}t_{2}}(1,1)+3A_{t_{1}t_{1}t_{2}}(1,1) +3A_{t_{2}t_{2}t_{1}}(1,1)\bigr\} , \\ \mathrm{(v)}\quad&K_{n}\bigl(t^{4};x\bigr)=x^{4}+ \frac{x^{3}}{n}\biggl\{ 6+\frac {4}{A(1,1)}\bigl(A_{t_{1}}(1,1)+A_{t_{2}}(1,1) \bigr)\biggr\} +\frac{x^{2}}{n^{2}}\biggl\{ 7\\ &\hphantom{K_{n}\bigl(t^{4};x\bigr)=}{}+\frac {6}{A(1,1)}\bigl(3A_{t_{1}}(1,1)+3A_{t_{2}}(1,1) +A_{t_{1}t_{1}}(1,1)+2A_{t_{1}t_{2}}(1,1)+A_{t_{2}t_{2}}(1,1)\bigr)\biggr\} \\ &\hphantom{K_{n}\bigl(t^{4};x\bigr)=}{}+\frac{x}{n^{3}}\biggl\{ 1+\frac{1}{A(1,1)}\bigl(14A_{t_{1}}(1,1)+14A_{t_{2}}(1,1)+ 18A_{t_{1}t_{1}}(1,1)+36A_{t_{1}t_{2}}(1,1)\\ &\hphantom{K_{n}\bigl(t^{4};x\bigr)=}{}+18A_{t_{2}t_{2}}(1,1) +4A_{t_{1}t_{1}t_{1}}(1,1)+4A_{t_{2}t_{2}t_{2}}(1,1)+12A_{t_{1}t_{1}t_{2}}(1,1)\\ &\hphantom{K_{n}\bigl(t^{4};x\bigr)=}{} +12A_{t_{2}t_{2}t_{1}}(1,1)\bigr)\biggr\} +\frac{1}{n^{4}A(1,1)}\bigl\{ A_{t_{1}}(1,1)+A_{t_{2}}(1,1)+7A_{t_{1}t_{1}}(1,1)\\ &\hphantom{K_{n}\bigl(t^{4};x\bigr)=}{}+14A_{t_{1}t_{2}}(1,1)+7A_{t_{2}t_{2}}(1,1)+6A_{t_{1}t_{1}t_{1}}(1,1)+6A_{t_{2}t_{2}t_{2}}(1,1)\\ &\hphantom{K_{n}\bigl(t^{4};x\bigr)=}{}+18A_{t_{1}t_{1}t_{2}}(1,1) +18A_{t_{2}t_{2}t_{1}}(1,1)+A_{t_{1}t_{1}t_{1}t_{1}}(1,1)+A_{t_{2}t_{2}t_{2}t_{2}}(1,1)\\ &\hphantom{K_{n}\bigl(t^{4};x\bigr)=}{} +4A_{t_{1}t_{1}t_{1}t_{2}}(1,1)+4A_{t_{2}t_{2}t_{2}t_{1}}(1,1)+6A_{t_{1}t_{1}t_{2}t_{2}}(1,1)\bigr\} . \end{aligned}$$

The values of the moments $K_{n}(t^{i};x)$ for $i=0,1,2$ are given in [10] while the values of $K_{n}(t^{i};x)$ for $i=3,4$ have been obtained by us after simple calculations and hence the details are omitted.

### Lemma 2.2

*For the sequence of linear positive operators* $\mathcal{L}_{n}^{\rho}(t^{i};x)$, $i=0,1,2,3,4$, *we find*

$$\begin{aligned} \mathrm{(i)}\quad&\mathcal{L}_{n}^{\rho}(1;x)=1, \\ \mathrm{(ii)}\quad&\mathcal{L}_{n}^{\rho}(t;x)=x+ \frac {A_{t_{1}}(1,1)+A_{t_{2}}(1,1)}{n A(1,1)}, \\ \mathrm{(iii)}\quad&\mathcal{L}_{n}^{\rho} \bigl(t^{2};x\bigr)=x^{2}+\frac{x}{n}\biggl\{ \biggl(1+ \frac{1}{\rho}\biggr)+\frac{2}{A(1,1)}\bigl(A_{t_{1}}(1,1)+A_{t_{2}}(1,1) \bigr)\biggr\} \\ &\hphantom{\mathcal{L}_{n}^{\rho} \bigl(t^{2};x\bigr)=}{} +\frac{1}{n^{2}A(1,1)}\biggl\{ \biggl(1+\frac{1}{\rho}\biggr) \bigl(A_{t_{1}}(1,1)+A_{t_{2}}(1,1)\bigr)+A_{t_{1}t_{1}}(1,1)\\ &\hphantom{\mathcal{L}_{n}^{\rho} \bigl(t^{2};x\bigr)=}{}+2A_{t_{1}t_{2}}(1,1)+A_{t_{2}t_{2}}(1,1) \biggr\} , \\ \mathrm{(iv)}\quad& \mathcal{L}_{n}^{\rho} \bigl(t^{3};x\bigr)=x^{3}+\frac{3x^{2}}{n}\biggl\{ \biggl(1+ \frac{1}{\rho}\biggr)+\frac{1}{A(1,1)}\bigl(A_{t_{1}}(1,1)+A_{t_{2}}(1,1) \bigr)\biggr\} \\ &\hphantom{\mathcal{L}_{n}^{\rho} \bigl(t^{3};x\bigr)=}{} +\frac{x}{n^{2}}\biggl\{ \biggl(1+\frac{3}{\rho}+ \frac{2}{\rho^{2}}\biggr) +\frac{3}{A(1,1)}\biggl(2\biggl(1+\frac{1}{\rho} \biggr) \bigl(A_{t_{1}}(1,1)+A_{t_{2}}(1,1)\bigr)\\ &\hphantom{\mathcal{L}_{n}^{\rho} \bigl(t^{3};x\bigr)=}{} +A_{t_{1}t_{1}}(1,1)+2A_{t_{1}t_{2}}(1,1)+A_{t_{2}t_{2}}(1,1)\biggr)\biggr\} \\ &\hphantom{\mathcal{L}_{n}^{\rho} \bigl(t^{3};x\bigr)=}{} +\frac{1}{n^{3}A(1,1)}\biggl\{ \biggl(1+\frac{3}{\rho}+\frac{2}{\rho ^{2}} \biggr) \bigl(A_{t_{1}}(1,1)+A_{t_{2}}(1,1)\bigr)\\ &\hphantom{\mathcal{L}_{n}^{\rho} \bigl(t^{3};x\bigr)=}{}+3\biggl(1+ \frac{1}{\rho}\biggr) \bigl(A_{t_{1}t_{1}}(1,1)+2A_{t_{1}t_{2}}(1,1) +A_{t_{2}t_{2}}(1,1)\bigr)+A_{t_{1}t_{1}t_{1}}(1,1) \\ &\hphantom{\mathcal{L}_{n}^{\rho} \bigl(t^{3};x\bigr)=}{}+A_{t_{2}t_{2}t_{2}}(1,1)+3A_{t_{1}t_{1}t_{2}}(1,1) +3A_{t_{2}t_{2}t_{1}}(1,1)\biggr\} , \\ \mathrm{(v)}\quad&\mathcal{L}_{n}^{\rho}\bigl(t^{4};x \bigr)=x^{4}+\frac{x^{3}}{n}\biggl\{ 6\biggl(1+\frac{1}{\rho} \biggr)+\frac{4}{A(1,1)}\bigl(A_{t_{1}}(1,1)+A_{t_{2}}(1,1)\bigr) \biggr\} \\ &\hphantom{\mathcal{L}_{n}^{\rho}\bigl(t^{4};x \bigr)=}{} +\frac{x^{2}}{n^{2}}\biggl\{ \biggl(7+\frac{18}{\rho}+ \frac{11}{\rho^{2}}\biggr) +\frac{6}{A(1,1)}\biggl(3\biggl(1+\frac{1}{\rho} \biggr) \bigl(A_{t_{1}}(1,1)+A_{t_{2}}(1,1)\bigr)\\ &\hphantom{\mathcal{L}_{n}^{\rho}\bigl(t^{4};x \bigr)=}{} +A_{t_{1}t_{1}}(1,1)+2A_{t_{1}t_{2}}(1,1)+A_{t_{2}t_{2}}(1,1)\biggr)\biggr\} +\frac{x}{n^{3}}\biggl\{ \biggl(1+\frac{6}{\rho}+\frac{11}{\rho^{2}}+ \frac{6}{\rho ^{3}}\biggr)\\ &\hphantom{\mathcal{L}_{n}^{\rho}\bigl(t^{4};x \bigr)=}{}+\frac{1}{A(1,1)} \biggl(\biggl(14+\frac{36}{\rho}+ \frac{22}{\rho^{2}}\biggr) \bigl(A_{t_{1}}(1,1)+A_{t_{2}}(1,1)\bigr)\\ &\hphantom{\mathcal{L}_{n}^{\rho}\bigl(t^{4};x \bigr)=}{} + 18\biggl(1+\frac{1}{\rho}\biggr) \bigl(A_{t_{1}t_{1}}(1,1)+2A_{t_{1}t_{2}}(1,1)+A_{t_{2}t_{2}}(1,1) \bigr) +4A_{t_{1}t_{1}t_{1}}(1,1)\\ &\hphantom{\mathcal{L}_{n}^{\rho}\bigl(t^{4};x \bigr)=}{}+4A_{t_{2}t_{2}t_{2}}(1,1) +12A_{t_{1}t_{1}t_{2}}(1,1)+ 12A_{t_{2}t_{2}t_{1}}(1,1)\biggr)\biggr\} \\ &\hphantom{\mathcal{L}_{n}^{\rho}\bigl(t^{4};x \bigr)=}{} +\frac{1}{n^{4}A(1,1)}\biggl\{ \biggl(1+ \frac{6}{\rho}+\frac{11}{\rho^{2}}+\frac{6}{\rho ^{3}}\biggr) \bigl(A_{t_{1}}(1,1)+A_{t_{2}}(1,1)\bigr)\\ &\hphantom{\mathcal{L}_{n}^{\rho}\bigl(t^{4};x \bigr)=}{} +\biggl(7+ \frac{18}{\rho}+\frac{11}{\rho ^{2}}\biggr) \bigl(A_{t_{1}t_{1}}(1,1)+2A_{t_{1}t_{2}}(1,1)+A_{t_{2}t_{2}}(1,1) \bigr)\\ &\hphantom{\mathcal{L}_{n}^{\rho}\bigl(t^{4};x \bigr)=}{} +6\biggl(1+\frac{1}{\rho}\biggr) \bigl(A_{t_{1}t_{1}t_{1}}(1,1)+A_{t_{2}t_{2}t_{2}}(1,1)+3A_{t_{1}t_{1}t_{2}}(1,1) +3A_{t_{2}t_{2}t_{1}}(1,1)\bigr)\\ &\hphantom{\mathcal{L}_{n}^{\rho}\bigl(t^{4};x \bigr)=}{} +A_{t_{1}t_{1}t_{1}t_{1}}(1,1)+A_{t_{2}t_{2}t_{2}t_{2}}(1,1) +4A_{t_{1}t_{1}t_{1}t_{2}}(1,1)\\ &\hphantom{\mathcal{L}_{n}^{\rho}\bigl(t^{4};x \bigr)=}{}+4A_{t_{2}t_{2}t_{2}t_{1}}(1,1)+6A_{t_{1}t_{1}t_{2}t_{2}}(1,1)\biggr\} . \end{aligned}$$

*Consequently*,

7 $$\begin{aligned} \mathcal{L}_{n}^{\rho}\bigl((t-x)^{2};x \bigr) =&\frac{x}{n}\biggl(1+\frac{1}{\rho}\biggr)+\frac {1}{n^{2}A(1,1)} \biggl\{ \biggl(1+\frac{1}{\rho}\biggr) \bigl(A_{t_{1}}(1,1)+A_{t_{2}}(1,1) \bigr) \\ &{}+A_{t_{1}t_{1}}(1,1)+2A_{t_{1}t_{2}}(1,1)+A_{t_{2}t_{2}}(1,1)\biggr\} \\ \leq&\frac{C}{n}\biggl(1+\frac{1}{\rho}\biggr) (1+x) \\ =&\delta_{n,\rho}^{2}(x) \quad (\textit{say}), \end{aligned}$$

*where*

$$C=\max\biggl(1, \frac{ \vert A_{t_{1}}(1,1) \vert + \vert A_{t_{2}}(1,1) \vert + \vert A_{t_{1},t_{1}}(1,1) \vert +2 \vert A_{t_{1},t_{2}}(1,1) \vert + \vert A_{t_{2},t_{2}}(1,1) \vert }{ \vert A(1,1) \vert } \biggr) $$

*and*

$$\begin{aligned} \mathcal{L}_{n}^{\rho}\bigl((t-x)^{4};x\bigr) =& \frac{3x^{2}}{n^{2}}\biggl\{ 1+\frac{2}{\rho}+\frac{1}{\rho^{2}}\biggr\} + \frac{x}{n^{3}}\biggl[\biggl(1+\frac{6}{\rho}+\frac{11}{\rho ^{2}}+ \frac{6}{\rho^{3}}\biggr)\\ &{} +\frac{1}{A(1,1)}\biggl\{ \biggl(13+\frac{33}{\rho}+ \frac{20}{\rho^{2}}\biggr) \bigl(A_{t_{1}}(1,1)+A_{t_{2}}(1,1)\bigr)\\ &{}+ 6\biggl(1+ \frac{1}{\rho}\biggr) \bigl(A_{t_{1}t_{1}}(1,1)+2A_{t_{1}t_{2}}(1,1)+A_{t_{2}t_{2}}(1,1) \bigr) \\ &{}-6A_{t_{1}t_{1}t_{1}}(1,1) -6A_{t_{2}t_{2}t_{2}}(1,1)\biggr\} \biggr]. \end{aligned}$$

The expression for $\mathcal{L}_{n}^{\rho}((t-x)^{6};x)$ has not been included in Lemma 2.2 because it is very lengthy and complicated. It will be required to prove the quantitative Voronovskaya type theorem.

### Remark 2.3

From Lemma 2.2, we obtain

8 $$\begin{aligned}& \lim_{n\rightarrow\infty}n\mathcal{L}_{n}^{\rho} \bigl((t-x);x\bigr) = \frac {A_{t_{1}}(1,1)+A_{t_{2}}(1,1)}{A(1,1)}, \end{aligned}$$

9 $$\begin{aligned}& \lim_{n\rightarrow\infty}n\mathcal{L}_{n}^{\rho} \bigl((t-x)^{2};x\bigr) = x\biggl(1+\frac {1}{\rho}\biggr), \end{aligned}$$

10 $$\begin{aligned}& \lim_{n \rightarrow\infty}n^{2} \mathcal{L}_{n}^{\rho} \bigl((t-x)^{4};x\bigr) = 3x^{2}\biggl(1+\frac{2}{\rho}+ \frac{1}{\rho^{2}}\biggr), \end{aligned}$$

11 $$\begin{aligned}& \lim_{n \rightarrow\infty}n^{3} \mathcal{L}_{n}^{\rho} \bigl((t-x)^{6};x\bigr) = 15x^{3}\biggl(1+\frac{3}{\rho}+ \frac{3}{\rho^{2}}+\frac{1}{\rho^{3}}\biggr). \end{aligned}$$

## Main results

### Theorem 3.1

*Let* $f\in C_{E}(R_{0}^{+})$. *Then* $\lim_{n\rightarrow\infty}\mathcal {L}_{n}^{\rho}(f;x)=f(x)$ *uniformly on each compact subset of* $R_{0}^{+}$.

### Proof

Considering Lemma 2.2, it follows that $\lim_{n\rightarrow\infty}\mathcal{L}_{n}^{\rho}(t^{i};x)=x^{i}$, $i=0,1,2$, uniformly on every compact subset of $R_{0}^{+}$. Applying the Bohman Korovkin theorem, we obtain the desired result. □

For $f\in C_{B}(R_{0}^{+})$, the space of bounded and continuous functions on $R_{0}^{+}$ endowed with the norm $\Vert f \Vert =\sup_{x\in R_{0}^{+}} \vert f(x) \vert $, the first and second order modulus of continuity are, respectively, defined as

$$\begin{gathered} \omega(f;\delta)=\sup_{0< \vert h \vert \leq\delta} \sup_{x,x+h\in R_{0}^{+}} \bigl\vert f(x+h)-f(x) \bigr\vert , \\ \omega_{2}(f;\delta)=\sup_{0< \vert h \vert \leq\delta} \sup _{x,x+h,x+2h\in R_{0}^{+}} \bigl\vert f(x+2h)-2f(x+h)+f(x) \bigr\vert , \quad \delta> 0. \end{gathered} $$

Further, for $f\in C_{B}(R_{0}^{+})$, the Steklov mean of second order [12] is defined as

12 $$\begin{aligned} f_{h}(x)= \frac{4}{h^{2}} \int_{0}^{\frac{h}{2}} \int_{0}^{\frac {h}{2}}\bigl[2f(x+u+v)-f\bigl(x+2(u+v)\bigr) \bigr]\,du\,dv, \quad h>0. \end{aligned}$$

Hence

$$\begin{aligned}& f(x)-f_{h}(x) = \frac{4}{h^{2}} \int_{0}^{h/2} \int_{0}^{h/2}\triangle ^{2}_{u+v}f(x)\,du\,dv,\quad \text{and} \\& f''_{h}(x) = \frac{1}{h^{2}}\bigl(8 \triangle^{2}_{h/2}f(x)-\triangle^{2}_{h}f(x) \bigr). \end{aligned}$$

Thus, it follows that

13 $$\begin{aligned} \Vert f_{h}-f \Vert \leq\omega_{2}(f,h). \end{aligned}$$

Further, $f_{h}^{\prime},f^{\prime\prime}_{h}\in C_{B}(R_{0}^{+})$ and

14 $$\begin{aligned} \bigl\Vert f^{\prime}_{h} \bigr\Vert \leq \frac{5}{h}\omega{(f,h)},\qquad \bigl\Vert f^{\prime\prime}_{h} \bigr\Vert \leq\frac{9}{h^{2}}\omega_{2}{(f,h)}. \end{aligned}$$

### Theorem 3.2

*For* $f\in C_{B}(R_{0}^{+})$ *and* $x \in R_{0}^{+}$, *we have*

$$\begin{aligned} \bigl\vert \mathcal{L}_{n}^{\rho}(f;x)-f(x) \bigr\vert \leq5\omega\bigl(f; \delta_{n,\rho}(x)\bigr)+\frac{13}{2} \omega_{2}\bigl(f;\delta_{n,\rho}(x)\bigr), \end{aligned}$$

*where* $\delta_{n,\rho}(x)$ *is defined by equation* (7).

### Proof

Using the Steklov mean $f_{h}$ defined by (12), we may write

15 $$\begin{aligned} { } \bigl\vert \mathcal{L}_{n}^{\rho}(f;x)-f(x) \bigr\vert \leq& \bigl\vert \mathcal{L}_{n}^{\rho}\bigl( (f-f_{h});x\bigr) \bigr\vert + \bigl\vert \mathcal {L}_{n}^{\rho} \bigl(f_{h}(t)-f_{h}(x);x \bigr) \bigr\vert \\ &{}+ \bigl\vert f_{h}(x)-f(x) \bigr\vert . \end{aligned}$$

Applying Lemma 2.2, we have

16 $$\begin{aligned} \bigl\Vert \mathcal{L}_{n}^{\rho}(f) \bigr\Vert \leq \Vert f \Vert , \end{aligned}$$

Using inequality (16) and equation (13), we have

$$\begin{aligned} \bigl\vert \mathcal{L}_{n}^{\rho}\bigl( (f-f_{h}) ;x\bigr) \bigr\vert \leq& \Vert f- f_{h} \Vert \\ \leq& \omega_{2}(f,h). \end{aligned}$$

Now by Taylor’s expansion and applying the Cauchy-Schwarz inequality, we have

$$\begin{aligned} \bigl\vert \mathcal{L}_{n}^{\rho}\bigl(f_{h}(t)-f_{h}(x);x \bigr) \bigr\vert \leq& \bigl\vert \mathcal{L}_{n}^{\rho} \bigl((t-x)f_{h}^{\prime}(x);x\bigr) \bigr\vert + \biggl\vert \mathcal{L}_{n}^{\rho}\biggl( \int_{x}^{t}(t-u)f_{h}^{\prime\prime}(u)\,du; x\biggr) \biggr\vert \\ \leq& \bigl\Vert f_{h}^{\prime} \bigr\Vert \bigl\vert \mathcal {L}_{n}^{\rho}\bigl( \vert t-x \vert ;x\bigr) \bigr\vert + \bigl\Vert f_{h}^{\prime\prime} \bigr\Vert \mathcal{L}_{n}^{\rho}\biggl( \biggl\vert \int_{x}^{t} \vert t-u \vert \,du \biggr\vert ;x\biggr) \\ =& \bigl\Vert f_{h}^{\prime} \bigr\Vert \sqrt { \mathcal{L}_{n}^{\rho}\bigl((t-x)^{2};x \bigr)} +\frac{1}{2} \bigl\Vert { f_{h}^{\prime\prime}} \bigr\Vert \mathcal{L}_{n}^{\rho}\bigl((t-x)^{2};x\bigr). \end{aligned}$$

Applying Lemma 2.2, equations (13), (14) and choosing *h* as $\delta_{n,\rho}(x)$, we get the required result. □

### Theorem 3.3

*For* $f\in C_{E}^{2}(R_{0}^{+})$, *we obtain*

$$\begin{aligned} \lim_{n\rightarrow\infty}n\bigl[\mathcal{L}_{n}^{\rho}(f;x)-f(x) \bigr]=\frac {A_{t_{1}}(1,1)+A_{t_{2}}(1,1)}{ A(1,1)}f'(x)+\frac{x}{2}\biggl(1+ \frac{1}{\rho}\biggr)f''(x), \end{aligned}$$

*uniformly in* $x\in[0,a]$, $a>0$.

### Proof

By Taylor’s expansion of *f* for some fixed $x\in[0,a]$, we obtain

17 $$\begin{aligned} f(t)-f(x)=(t-x)f'(x)+\frac{1}{2}(t-x)^{2}f''(x)+ \xi(t,x) (t-x)^{2}, \end{aligned}$$

where $\xi(t,x)\in C_{E}(R_{0}^{+})$ and $\lim_{t\rightarrow x}\xi(t,x)= 0$.

Hence by linearity of the operators $\mathcal{L}_{n}^{\rho}$, from equation (17), we get

18 $$\begin{aligned} n\bigl[\mathcal{L}_{n}^{\rho}(f;x)-f(x)\bigr] =& n \mathcal{L}_{n}^{\rho}(t-x;x)f'(x)+ \frac{1}{2} n \mathcal{L}_{n}^{\rho}\bigl((t-x)^{2};x \bigr)f''(x) \\ &{}+n \mathcal{L}_{n}^{\rho}\bigl(\xi(t,x) (t-x)^{2};x\bigr). \end{aligned}$$

Applying the Cauchy-Schwarz inequality in the last term of equation (18), we have

19 $$\begin{aligned} n \mathcal{L}_{n}^{\rho}\bigl(\xi(t,x) (t-x)^{2};x\bigr)\leq\sqrt{ n^{2}\mathcal {L}_{n}^{\rho}\bigl((t-x)^{4};x\bigr) \mathcal{L}_{n}^{\rho}\bigl(\xi^{2}(t,x);x\bigr)}. \end{aligned}$$

From Remark 2.3, it follows that

20 $$\begin{aligned} \lim_{n\rightarrow\infty}{n^{2}} \mathcal{L}_{n}^{\rho}\bigl((t-x)^{4};x \bigr)=3x^{2}\biggl(1+\frac{2}{\rho}+\frac{1}{\rho^{2}}\biggr), \end{aligned}$$

uniformly in $x\in[0,a]$.

Further, let $\xi^{2}(t,x)=\nu(t,x)$, $x\geq0$, then $\nu(t,x)\in C_{E}(R_{0}^{+})$ and hence from Theorem 1.1, we get

21 $$\begin{aligned} \lim_{n\rightarrow\infty}\mathcal{L}_{n}^{\rho} \bigl(\xi^{2}(t,x);x\bigr)=\lim_{n\rightarrow\infty} \mathcal{L}_{n}^{\rho}\bigl(\nu(t,x);x\bigr)=\nu(x,x)=0, \end{aligned}$$

Hence from equation (19), we obtain

22 $$\begin{aligned} \lim_{n\rightarrow\infty}\bigl(n \mathcal{L}_{n}^{\rho} \bigl(\xi(t,x) (t-x)^{2};x\bigr)\bigr)=0, \end{aligned}$$

uniformly in $x\in[0,a]$. Now taking the limit $n\rightarrow\infty$ in (18) and using Remark 2.3, we get the desired result. This completes the proof. □

## Weighted approximation

Let $\theta(x)\geq1$ be a weight function on $R_{0}^{+}$. We consider the weighted space defined on $R^{+}_{0}$:

$$B_{\theta}\bigl(R^{+}_{0}\bigr):=\bigl\{ f: \bigl\vert f(x) \bigr\vert \leq M_{f}\theta(x), \forall x \in R_{0}^{+} \mbox{ and } M_{f} > 0\bigr\} . $$

Further, let

$$C_{\theta}\bigl(R^{+}_{0}\bigr):=\bigl\{ f\in B_{\theta} \bigl(R^{+}_{0}\bigr):f \text{ is a continuous function on }R_{0}^{+}\bigr\} $$

and

$$C^{*}_{\theta}\bigl(R^{+}_{0}\bigr):=\biggl\{ f\in C_{\theta}\bigl(R^{+}_{0}\bigr): \lim_{x\rightarrow\infty} \frac{f(x)}{\theta(x)}=K_{f}< \infty\biggr\} . $$

We define the norm in the space $B_{\theta}(R^{+}_{0})$ as

$$\Vert f \Vert _{\theta}=\sup_{x\in R^{+}_{0}} \frac{ \vert f(x) \vert }{\theta(x)}. $$

The usual modulus of continuity of the function *f* on $[0,p]$ is defined as

23 $$\begin{aligned} \omega_{p}(f;\delta)=\sup_{ \vert t-x \vert \leq\delta}\sup _{t, x \in[0,p]} \bigl\vert f(t)-f(x) \bigr\vert . \end{aligned}$$

Let us denote $\Vert \cdot \Vert _{C[a,b]}$ as the supremum norm on $[a,b]$. Throughout the paper we have taken $\theta(x)=1+x^{2}$.

### Theorem 4.1

*For* $x\in[0,c]$ *and* $f\in C_{\theta}(R_{0}^{+})$, *we have*

$$\begin{aligned} \bigl\Vert \mathcal{L}_{n}^{\rho}(f;\cdot)-f \bigr\Vert _{C[0,c]} \leq&4 M_{f}\bigl(1+c^{2}\bigr) \eta_{n,\rho}^{2} +2\omega_{c+1}(f;\eta_{n,\rho} ), \end{aligned}$$

*where* $\eta^{2}_{n, \rho}=\max_{x \in[0,c]}(\mathcal{L}_{n}^{\rho}((t-x^{2});x))$.

### Proof

Let $x\in[0,c]$ and $t>c+1$ then $t-x> 1$. Then, for $f\in C_{\theta}(R_{0}^{+})$, we have

24 $$\begin{aligned} \bigl\vert f(t)-f(x) \bigr\vert &\leq M_{f} \bigl(2+t^{2}+x^{2}\bigr) \\ &=M_{f}\bigl(2+2x^{2}+(t-x)^{2}+2x(t-x)\bigr) \\ &\leq M_{f}(t-x)^{2}\bigl(3+2x^{2}+2x\bigr) \leq4 M_{f}\bigl(1+x^{2}\bigr) (t-x)^{2}. \end{aligned}$$

For $x\in[0,c]$ and $t\in[0,c+1]$, we have

25 $$\begin{aligned} \bigl\vert f(t)-f(x) \bigr\vert \leq\omega_{c+1} \bigl(f; \vert t-x \vert \bigr)\leq\biggl(1+\frac{ \vert t-x \vert }{\delta}\biggr)\omega _{c+1}(f;\delta). \end{aligned}$$

From equations (24) and (25), for $x\in[0,c]$ and $t\geq 0$, we have

$$\begin{aligned} \bigl\vert f(t)-f(x) \bigr\vert \leq4M_{f}\bigl(1+x^{2} \bigr) (t-x)^{2}+\biggl(1+\frac{ \vert t-x \vert }{\delta}\biggr)\omega_{c+1}(f; \delta). \end{aligned}$$

Applying the Cauchy-Schwarz inequality and choosing $\delta=\sqrt{\eta _{n,\rho}}$, we get

$$\begin{aligned} \bigl\vert \mathcal{L}_{n}^{\rho}(f;x)-f(x) \bigr\vert & \leq 4M_{f}\bigl(1+x^{2}\bigr)\mathcal{L}_{n}^{\rho} \bigl(\bigl(t-x^{2}\bigr);x\bigr)+\biggl(1+\frac{1}{\delta}\mathcal {L}_{n}^{\rho}\bigl( \vert t-x \vert ;x\bigr)\biggr) \omega_{c+1}(f;\delta) \\ &\leq4M_{f}\bigl(1+c^{2}\bigr)\eta_{n,\rho}^{2}(c) +2\omega_{c+1}\bigl(f;\eta_{n,\rho}(c) \bigr). \end{aligned}$$

This completes the proof. □

### Theorem 4.2

*For* $f\in C_{\theta}(R_{0}^{+})$, *we have*

$$\begin{aligned} \lim_{n\rightarrow\infty}\sup_{x\in R_{0}^{+}}\frac{ \vert \mathcal {L}_{n}^{\rho}(f;x)-f(x) \vert }{(1+x^{2})^{1+\eta}}=0, \end{aligned}$$

*where* *η* *is some positive constant*.

### Proof

Since $\vert f(x) \vert \leq \Vert f \Vert _{\theta}(1+x^{2})$, for fixed $y > 0$, we may write

26 $$\begin{aligned} \sup_{x\in R_{0}^{+}}\frac{ \vert \mathcal{L}_{n}^{\rho}(f;x)-f(x) \vert }{(1+x^{2})^{1+\eta}}&\leq\sup _{x\in[0,y]}\frac{ \vert \mathcal {L}_{n}^{\rho}(f;x)-f(x) \vert }{(1+x^{2})^{1+\eta}}+ \sup_{x\in(y,\infty)} \frac{ \vert \mathcal{L}_{n}^{\rho}(f;x)-f(x) \vert }{(1+x^{2})^{1+\eta}} \\ &\leq \bigl\Vert \mathcal{L}_{n}^{\rho}(f; \cdot)-f \bigr\Vert _{C[0,y]}+\frac { \Vert f \Vert _{\theta}}{(1+y^{2})^{\eta}} \\ &\quad {}+ \Vert f \Vert _{\theta}\sup_{x\in(y,\infty)} \frac{ \vert \mathcal{L}_{n}^{\rho}(1+t^{2};x) \vert }{(1+x^{2})^{1+\eta}}. \end{aligned}$$

Using Theorem 3.1, for a given $\epsilon>0$, there exists $k \in\mathbb{N}$ such that

$$\begin{aligned} \bigl\vert \mathcal{L}_{n}^{\rho}\bigl(1+t^{2};x \bigr)-{1+x^{2}} \bigr\vert < \frac {\epsilon}{3 \Vert f \Vert _{\theta}}, \quad \forall n\geq k. \end{aligned}$$

or

$$\begin{aligned} \mathcal{L}_{n}^{\rho}\bigl(1+t^{2};x \bigr)< {1+x^{2}}+\frac{\epsilon}{3 \Vert f \Vert _{\theta}}, \quad \forall n\geq k. \end{aligned}$$

Hence,

$$\begin{aligned} \Vert f \Vert _{\theta}\frac{\mathcal{L}_{n}^{\rho}(1+t^{2};x)}{(1+x^{2})^{1+\eta}} < & \frac{ \Vert f \Vert _{\theta}}{(1+x^{2})^{1+\eta}} \biggl(1+x^{2}+\frac{\epsilon}{3 \Vert f \Vert _{\theta}}\biggr) \\ < &\frac{ \Vert f \Vert _{\theta}}{(1+y^{2})^{\eta}}+\frac {\epsilon}{3} ,\quad \forall n\geq k. \end{aligned}$$

Therefore,

27 $$\begin{aligned} \Vert f \Vert _{\theta}\sup_{x\in[y,\infty)} \frac{\mathcal {L}_{n}^{\rho}(1+t^{2};x)}{(1+x^{2})^{1+\eta}}\leq\frac{ \Vert f \Vert _{\theta}}{(1+y^{2})^{\eta}}+\frac{\epsilon}{3},\quad \text{for all } n \geq k. \end{aligned}$$

Let us choose y so large that

28 $$\begin{aligned} \frac{ \Vert f \Vert _{\theta}}{(1+y^{2})^{\eta}}\leq\frac {\epsilon}{6}. \end{aligned}$$

Also, in view of Theorem 4.1, for $\epsilon>0$ there exists a $n\geq l$ such that

29 $$ \bigl\Vert \mathcal{L}_{n}^{\rho}(f; \cdot)-f \bigr\Vert _{C[0,y]}< \frac {\epsilon}{3}, \quad n\geq l. $$

Taking $m=\max(k, l)$ and combining equations (26)-(29), we get

$$\sup_{x\in R_{0}^{+}}\frac{ \vert \mathcal{L}_{n}^{\rho}(f;x)-f(x) \vert }{(1+x^{2})^{1+\eta}}< \epsilon,\quad n\geq m. $$

This completes the proof. □

Following [13], the weighted modulus of continuity $\overline {\omega}(g;\delta)$ for $g\in C_{\theta}(R_{0}^{+})$ is defined as

30 $$\begin{aligned} \overline{\omega}(g;\delta)=\sup_{0< \vert h \vert \leq\delta , x\in R_{0}^{+}} \frac{ \vert g(x+h)-g(x) \vert }{(1+h^{2})(1+x^{2})}. \end{aligned}$$

Also, for $g\in C^{*}_{\theta}(R_{0}^{+})$, the weighted modulus of continuity has the following properties:

$$\begin{aligned} \lim_{\delta\rightarrow0}\overline{\omega}(g;\delta)=0 \end{aligned}$$

and

31 $$\begin{aligned} \overline{\omega}(g;\lambda\delta)\leq2(1+\lambda) \bigl(1+ \delta^{2}\bigr)\overline {\omega}(g;\delta),\quad \lambda> 0. \end{aligned}$$

For $g\in C_{\theta}(R_{0}^{+})$, from equations (30) and (31)

32 $$\begin{aligned} \bigl\vert g(t)-g(x) \bigr\vert \leq& \bigl(1+(t-x)^{2} \bigr) \bigl(1+x^{2}\bigr)\overline {\omega}\bigl(g; \vert t-x \vert \bigr) \\ \leq&2\biggl(1+\frac{ \vert t-x \vert }{\delta}\biggr) \bigl(1+\delta ^{2}\bigr) \overline{\omega}(g;\delta) \bigl(1+(t-x)^{2}\bigr) \bigl(1+x^{2}\bigr). \end{aligned}$$

### Theorem 4.3

*For* $f\in C_{\theta}^{*}(R_{0}^{+})$, *we have*

$$\begin{aligned} \sup_{x\in R_{0}^{+}}\frac{ \vert \mathcal{L}_{n}^{\rho}(f;x)-f(x) \vert }{(1+x^{2})^{2}}\leq C\overline{\omega}\biggl(f; \sqrt{\frac{1}{n}}\biggr), \end{aligned}$$

*where C is a positive constant independent of* *n*.

### Proof

By the linearity and positivity of the operators $\mathcal{L}_{n}^{\rho}$, we get

$$\begin{aligned} \bigl\vert \mathcal{L}_{n}^{\rho}(f;x)-f(x) \bigr\vert \leq\mathcal {L}_{n}^{\rho}\bigl( \bigl\vert f(t)-f(x) \bigr\vert ;x\bigr) \end{aligned}$$

Using equation (32) and the Cauchy-Schwarz inequality, we get

33 $$ \begin{aligned}[b] \bigl\vert \mathcal{L}_{n}^{\rho}(f;x)-f(x) \bigr\vert &\leq 2\bigl(1+\delta ^{2}\bigr)\overline{\omega}(f; \delta) \bigl(1+x^{2}\bigr)\mathcal{L}_{n}^{\rho} \biggl(\biggl(1+\frac{ \vert t-x \vert }{\delta}\biggr) \bigl(1+(t-x)^{2}\bigr);x \biggr) \\ &\leq 2\bigl(1+\delta^{2}\bigr)\overline{\omega}(f;\delta) \bigl(1+x^{2}\bigr)\biggl\{ \mathcal {L}_{n}^{\rho}(1;x)+ \mathcal{L}_{n}^{\rho}\bigl((t-x)^{2};x\bigr) \\ &\quad {}+\frac{1}{\delta}\mathcal{L}_{n}^{\rho}\bigl( \vert t-x \vert ;x\bigr) +\frac{1}{\delta}\mathcal{L}_{n}^{\rho} \bigl( \vert t-x \vert (t-x)^{2};x\bigr)\biggr\} \\ &\leq 2\bigl(1+\delta^{2}\bigr)\overline{\omega}(f;\delta) \bigl(1+x^{2}\bigr)\biggl\{ \mathcal {L}_{n}^{\rho}(1;x)+ \mathcal{L}_{n}^{\rho}\bigl((t-x)^{2};x\bigr) \\ &\quad {}+\frac{1}{\delta}\sqrt{\mathcal{L}_{n}^{\rho} \bigl((t-x)^{2};x\bigr)} +\frac{1}{\delta}\sqrt{ \mathcal{L}_{n}^{\rho}\bigl((t-x)^{2};x\bigr)}\sqrt {\mathcal {L}_{n}^{\rho}\bigl((t-x)^{4};x \bigr)}\biggr\} . \end{aligned} $$

Using Lemma 2.2, we obtain

34 $$\begin{aligned} \mathcal{L}_{n}^{\rho}\bigl((t-x)^{2};x \bigr)\leq C_{1}\frac{1}{n}\bigl(1+x^{2}\bigr) \end{aligned}$$

and

35 $$\begin{aligned} \mathcal{L}_{n}^{\rho}\bigl((t-x)^{4};x \bigr)\leq C_{2}\frac{1}{n^{2}}\bigl(1+x^{2}\bigr), \end{aligned}$$

for some positive constants $C_{1}$ and $C_{2}$ dependent on *ρ* and $A(t_{1},t_{2})$. Now combining equations (33)-(35) and taking $\delta=\sqrt {\frac{1}{n}}$, we have

$$\begin{aligned} \bigl\vert \mathcal{L}_{n}^{\rho}(f;x)-f(x) \bigr\vert \leq& 2\biggl(1+\frac {1}{n}\biggr)\overline{\omega}\biggl(f;\sqrt{ \frac{1}{n}}\biggr) \bigl(1+x^{2}\bigr)\biggl\{ 1+C_{1} \frac {1}{n}\bigl(1+x^{2}\bigr) \\ &{}+\sqrt{C_{1}}\sqrt{\bigl(1+x^{2}\bigr)}+ \sqrt{C_{1}}\sqrt{\bigl(1+x^{2}\bigr)}\sqrt{C_{2}} \sqrt {\bigl(1+x^{2}\bigr)}\biggr\} . \end{aligned}$$

Hence, we get

$$\begin{aligned} \sup_{x\in R_{0}^{+}}\frac{ \vert \mathcal{L}_{n}^{\rho}(f;x)-f(x) \vert }{(1+x^{2})^{2}}\leq C\overline{\omega}\biggl(f; \sqrt{\frac{1}{n}}\biggr), \end{aligned}$$

where $C=2(1+MC_{1}+\sqrt{C_{1}}+\sqrt{C_{1}}\sqrt{C_{2}})$. This completes the proof. □

## Quantitative Voronovskaya theorems

In the following result, we discuss a quantitative Voronovskaja type theorem by using the weighted modulus of smoothness $\overline{\omega}(f;\delta)$. Recently, many researchers [14–18] have made remarkable contributions in this area.

### Theorem 5.1

*For* *f*, $f^{\prime}$, $f^{\prime\prime}$ *in* $C_{\theta}^{*}(R_{0}^{+})$ *and any* $x\in R_{0}^{+}$, *we have*

$$\begin{aligned}& \biggl\vert n\bigl(\mathcal{L}_{n}^{\rho}(f ;x)-f(x) \bigr)-f^{\prime}(x) \biggl(\frac {A_{t_{1}}(1,1)+A_{t_{2}}(1,1)}{ A(1,1)}\biggr) -\frac{f^{\prime\prime}(x)}{2!} \biggl[\frac{x}{n}\biggl(1+\frac{1}{\rho}\biggr) \\& \qquad {}+\frac{1}{n^{2}A(1,1)}\biggl\{ \biggl(1+\frac{1}{\rho}\biggr) \bigl(A_{t_{1}}(1,1)+A_{t_{2}}(1,1)\bigr)\\& \qquad {}+A_{t_{1}t_{1}}(1,1)+2A_{t_{1}t_{2}}(1,1)+A_{t_{2}t_{2}}(1,1) \biggr\} \biggr]\biggr\vert \\& \quad =O(1) \overline{\omega} \biggl(f^{\prime\prime};\frac{1}{\sqrt{n}}\biggr),\quad \textit{as }n\rightarrow\infty. \end{aligned}$$

### Proof

Let $x,t\in R_{0}^{+}$, then, by Taylor’s expansion, we have

$$\begin{aligned} f(t)=f(x)+f'(x) (t-x)+\frac{f^{\prime\prime}(x)}{2!}(t-x)^{2}+ E(t,x), \end{aligned}$$

where $E(t, x)=\frac{f^{\prime\prime}(\varphi)-f^{\prime\prime}(x)}{2!}(t-x)^{2}$ and *φ* lies between *t* and *x*.

Now, we get

$$\begin{aligned} \biggl\vert \mathcal{L}_{n}^{\rho}(f;x)-f(x)-f^{\prime}(x) \mathcal{L}_{n}^{\rho}\bigl((t-x);x\bigr) -\frac{f^{\prime\prime}(x)}{2!} \mathcal{L}_{n}^{\rho}\bigl((t-x)^{2};x\bigr)\biggr\vert \leq \mathcal{L}_{n}^{\rho}\bigl( \bigl\vert E(t,x) \bigr\vert ;x\bigr). \end{aligned}$$

Multiplying by *n* on both sides of the above inequality and using Lemma 2.2, we obtain

36 $$\begin{aligned}& \biggl\vert n\bigl(\mathcal{L}_{n}^{\rho}(f ;x)-f(x) \bigr)-f^{\prime}(x) \biggl(\frac {A_{t_{1}}(1,1)+A_{t_{2}}(1,1)}{ A(1,1)}\biggr) -\frac{f^{\prime\prime}(x)}{2!} \biggl[\frac{x}{n}\biggl(1+\frac{1}{\rho}\biggr) \\& \qquad {}+\frac{1}{n^{2}A(1,1)}\biggl\{ \biggl(1+\frac{1}{\rho}\biggr) \bigl(A_{t_{1}}(1,1)+A_{t_{2}}(1,1)\bigr) \\& \qquad {}+A_{t_{1}t_{1}}(1,1)+2A_{t_{1}t_{2}}(1,1)+A_{t_{2}t_{2}}(1,1) \biggr\} \biggr]\biggr\vert \\& \quad \leq n\mathcal{L}_{n}^{\rho}\bigl( \bigl\vert E(t,x) \bigr\vert ;x\bigr). \end{aligned}$$

Using the property of weighted modulus of smoothness given by (32), we get

$$\begin{aligned} \biggl\vert \frac{f^{\prime\prime}(\varphi)-f^{\prime\prime}(x)}{2!} \biggr\vert \leq& \frac{1}{2}\overline{\omega}\bigl(f^{\prime\prime}; \vert \varphi-x \vert \bigr) \bigl(1+(\varphi-x)^{2}\bigr) \bigl(1+x^{2} \bigr) \\ \leq&\frac{1}{2}\overline{\omega}\bigl(f^{\prime\prime}; \vert t-x \vert \bigr) \bigl(1+(t-x)^{2}\bigr) \bigl(1+x^{2}\bigr) \\ \leq& \biggl(1+\frac{( \vert t-x \vert )}{\delta}\biggr) \bigl(1+\delta ^{2}\bigr) \overline{\omega}\bigl(f^{\prime\prime};\delta\bigr) \\ &\times\bigl(1+(t-x)^{2}\bigr) \bigl(1+x^{2}\bigr). \end{aligned}$$

Also,

$$\biggl\vert \frac{f^{\prime\prime}(\varphi)-f^{\prime\prime}(x)}{2!} \biggr\vert \leq \textstyle\begin{cases} 2(1+\delta^{2})^{2}(1+x^{2})\overline{\omega}(f^{\prime\prime};\delta), & \vert t-x \vert \leq\delta,\\ 2(1+\delta^{2})^{2}(1+x^{2})\frac{(t-x)^{2}}{\delta^{4}}\overline{\omega}(f^{\prime\prime};\delta), & \vert t-x \vert \geq\delta. \end{cases} $$

Now for $0<\delta<1$, we obtain

$$\begin{aligned} \biggl\vert \frac{f^{\prime\prime}(\varphi)-f^{\prime\prime}(x)}{2!} \biggr\vert \leq& 8\bigl(1+x^{2} \bigr)\overline{\omega}\bigl(f^{\prime\prime};\delta\bigr) \biggl(1+ \frac {(t-x)^{4}}{\delta^{4}}\biggr). \end{aligned}$$

Therefore, we get

$$\begin{aligned} \bigl\vert E(t,x) \bigr\vert \leq& 8\bigl(1+x^{2}\bigr)\overline{ \omega}\bigl(f^{\prime \prime};\delta\bigr) \biggl( (t-x)^{2}+ \frac{(t-x)^{6}}{\delta^{4}}\biggr). \end{aligned}$$

Now by the linearity and positivity of the operator $\mathcal{L}_{n}^{\rho}$ and using Remark 2.3, for any $x\in R_{0}^{+}$, we obtain

$$\begin{aligned} \mathcal{L}_{n}^{\rho}\bigl( \bigl\vert E(t,x) \bigr\vert ;x\bigr) \leq& 8\bigl(1+x^{2}\bigr)\overline{\omega} \bigl(f^{\prime\prime};\delta\bigr)\biggl\{ \mathcal{L}_{n}^{\rho} \bigl((t-x)^{2};x\bigr) +\frac{1}{\delta^{4}} \mathcal{L}_{n}^{\rho} \bigl((t-x)^{6};x\bigr)\biggr\} \\ \leq& 8\bigl(1+x^{2}\bigr)\overline{\omega}\bigl(f^{\prime\prime}; \delta\bigr)\biggl\{ O\biggl(\frac {1}{n}\biggr)+O\biggl(\frac{1}{n^{3}} \biggr)\biggr\} . \end{aligned}$$

Choosing $\delta=\frac{1}{\sqrt{n}}$, we obtain

37 $$\begin{aligned} \mathcal{L}_{n}^{\rho}\bigl( \bigl\vert E(t,x) \bigr\vert ;x\bigr) \leq &8\bigl(1+x^{2}\bigr)\overline{\omega} \bigl(f^{\prime\prime};n^{-\frac{1}{2}}\bigr)O\biggl(\frac{1}{n}\biggr). \end{aligned}$$

Hence combining (36) and (37), we reach the required result. □

## Grüss-Voronovskaya-type theorem

For the first time Gal and Gonska [19], studied the Grüss Voronovskaya type theorem for the Bernstein, Păltănea and Bernstein-Faber operators by means of the Grüss inequality which concerns the non-multiplicavity of these operators. For more papers in this direction we refer the reader to (*cf.* [20–22] etc.) Next, we study the non-multiplicativity of the positive linear operator $\mathcal{L}_{n}^{\rho}$.

### Theorem 6.1

*For* $f^{\prime}(x), g^{\prime}(x), f^{\prime\prime}(x), g^{\prime \prime}(x), (fg)^{\prime}(x), (fg)^{\prime\prime}(x)\in C_{\theta}^{*}(R_{0}^{+})$, *we have the following equality*:

$$\begin{aligned} \lim_{n\rightarrow\infty}n\bigl\{ \mathcal{L}_{n}^{\rho}(fg;x)- \mathcal {L}_{n}^{\rho}(f;x)\mathcal{L}_{n}^{\rho}(g;x) \bigr\} =x\biggl(1+\frac{1}{\rho}\biggr)f^{\prime}(x)g^{\prime}(x). \end{aligned}$$

### Proof

We have

$$\begin{aligned} (fg)^{\prime\prime}(x)=f^{\prime\prime}(x)g(x)+ 2f^{\prime}(x)g^{\prime}(x)+ g^{\prime\prime}(x)f(x). \end{aligned}$$

By making an appropriate arrangement, we get

$$\begin{gathered} n\bigl\{ \mathcal{L}_{n}^{\rho}\bigl((fg);x\bigr)- \mathcal{L}_{n}^{\rho}(f;x)\mathcal {L}_{n}^{\rho}(g;x) \bigr\} \\ \quad = n\biggl\{ \mathcal{L}_{n}^{\rho}\bigl((fg);x \bigr)-f(x)g(x)-(fg)^{\prime}(x)\mathcal{L}_{n}^{\rho}(t-x;x)- \frac{(fg)^{\prime\prime}(x)}{2!}\mathcal{L}_{n}^{\rho}\bigl((t-x)^{2};x \bigr) \\ \qquad {}-g(x) \bigl(\mathcal{L}_{n}^{\rho}(f;x)-f(x) -f^{\prime}(x)\bigr)\mathcal{L}_{n}^{\rho}(t-x;x) - \frac{f^{\prime\prime}(x)}{2!}\mathcal{L}_{n}^{\rho}\bigl((t-x)^{2};x \bigr) \\ \qquad {}-\mathcal{L}_{n}^{\rho}(f;x) \bigl( \mathcal{L}_{n}^{\rho}(g;x)-g(x) -(g)^{\prime}(x)\bigr) \mathcal{L}_{n}^{\rho}(t-x;x)-\frac{(g)^{\prime\prime}(x)}{2!} \mathcal{L}_{n}^{\rho}\bigl((t-x)^{2};x\bigr) \\ \qquad {}+2\frac{\mathcal{L}_{n}^{\rho}((t-x)^{2};x)}{2!}f^{\prime}(x)) (g)^{\prime}(x))+(g)^{\prime\prime}(x)) \frac{\mathcal{L}_{n}^{\rho}((t-x)^{2};x)}{2!}\bigl(f(x) -\mathcal{L}_{n}^{\rho}(f;x)\bigr) \\ \qquad {}+(g)^{\prime}(x))\mathcal{L}_{n}^{\rho}(t-x;x) \bigl(f(x)-\mathcal {L}_{n}^{\rho}(f;x)\bigr)\biggr\} . \end{gathered} $$

Applying Theorem 3.1, for each $x\in R_{0}^{+}$, $L_{n}^{\rho}(f;x)\rightarrow f(x)$ as $n\rightarrow\infty$ and for $f^{\prime\prime}\in C_{\theta}^{*}(R_{0}^{+})$, $x\in R_{0}^{+}$, by Theorem 5.1, we have

$$\lim_{n\rightarrow\infty}\biggl(\mathcal{L}_{n}^{\rho}(f;x)-f(x)-f^{\prime}(x) \mathcal{L}_{n}^{\rho}\bigl((t-x);x\bigr) -\frac{f^{\prime\prime}(x)}{2!} \mathcal{L}_{n}^{\rho}\bigl((t-x)^{2};x\bigr)\biggr)= 0. $$

Therefore, using Remark 2.3, we get the desired result. □

## Rate of approximation of functions having derivative of bounded variation

In the last decade, the degree of approximation for the functions having a derivative of bounded variation has been studied by several researchers. Ispir *et al.* [23] considered the Kantorovich modification of Lupas operators based on Polya distributions and studied the rate of approximation of the functions having a derivative of bounded variation. For other significant contributions in this direction *cf.* [16, 24–27] etc. Motivated by these studies, we shall discuss the rate of approximation of functions with a derivative of bounded variation on $R_{0}^{+}$ for the operators $\mathcal{L}_{n}^{\rho}$.

Let $\operatorname{DBV}(R_{0}^{+})$ be the subspace of $B_{\theta}(R_{0}^{+})$ of all absolutely continuous functions *f* having a derivative $f^{\prime}$ equivalent with a function of bounded variation on every finite subinterval of $R_{0}^{+}$. We observe that the functions $f \in \operatorname{DBV}(R_{0}^{+})$ possess a representation

$$\begin{aligned} f(x)= \int_{0}^{x}g(t)\,dt+f(0), \end{aligned}$$

where $g\in BV(R_{0}^{+})$, *i.e.*, *g* is a function of bounded variation on every finite subinterval of $R_{0}^{+}$.

In order to discuss the approximation of functions with derivatives of bounded variation, we express the operators $\mathcal{L}_{n}^{\rho}$ in an integral form as follows:

38 $$\begin{aligned} \mathcal{L}_{n}^{\rho}(f;x)= \int_{0}^{\infty}K_{n}^{\rho}( x,t) f(t) \,dt, \end{aligned}$$

where the kernel $K_{n}^{\rho}(x,t)$ is given by

$$\begin{aligned} K_{n}^{\rho}(x,t)&=\frac{e^{-nx}}{A(1,1)}{\sum _{k_{1}}}_{k_{1}+k_{2}\geq1}\sum_{k_{2}} \frac{p_{k_{1},k_{2}}(\frac{n x}{2})}{{k_{1}}!{k_{2}}!}\frac{n\rho e^{-n\rho t}(n\rho t)^{(k_{1}+k_{2})\rho-1}}{\Gamma{(k_{1}+k_{2})\rho}}\\ &\quad {}+\frac {e^{-nx}}{A(1,1)}p_{0,0}\biggl( \frac{n x}{2}\biggr)\delta(t), \end{aligned} $$

$\delta(t)$ being the Dirac-delta function.

### Lemma 7.1

*For a fixed* $x\in R_{0}^{+}$ *and sufficiently large* *n*, *we have*

$$\begin{aligned} \mathrm{(i)}\quad &\xi_{n}^{\rho}(x,y)=\int_{0}^{y}K_{n}^{\rho}(x,t)\,dt\leq \frac {C(1+\frac{1}{\rho})(1+x)}{n}\frac{1}{(x-y)^{2}},\quad 0\leq y< x, \\ \mathrm{(ii)}\quad &1-\xi_{n}^{\rho}(x,z)=\int_{z}^{\infty}K_{n}^{\rho}(x,t)\,dt\leq\frac {C(1+\frac{1}{\rho})(1+x)}{n}\frac{1}{(z-x)^{2}},\quad x< z< \infty. \end{aligned}$$

### Proof

(i) Using Lemma 2.2, we get

$$\begin{aligned} \xi_{n}^{\rho}(x,y) =& \int _{0}^{y}K_{n}^{\rho}(x,t)\,dt\leq \int _{0}^{y}\biggl(\frac{x-t}{x-y} \biggr)^{2}K_{n}^{\rho}(x,t)\,dt \\ \leq& \mathcal{L}_{n}^{\rho}\bigl((t-x)^{2};x \bigr) ( x-y) ^{-2} \\ \leq&\frac{C(1+\frac{1}{\rho})(1+x)}{n}\frac{1}{(x-y)^{2}}. \end{aligned}$$

The proof of (ii) is similar; hence the details are omitted. □

### Theorem 7.2

*Let* $f \in \operatorname{DBV}(R_{0}^{+})$. *Then*, *for every* $x\in R_{0}^{+}$ *and sufficiently large* *n*, *we have*

$$\begin{aligned}& \bigl\vert \mathcal{L}_{n}^{\rho}(f;x)-f(x) \bigr\vert \\& \quad \leq\frac{1}{2}\bigl( f^{\prime}(x+)+f^{\prime}(x-) \bigr) \biggl(\frac {A_{t_{1}}(1,1)+A_{t_{2}}(1,1)}{n A(1,1)}\biggr)+\frac{1}{2} \bigl\vert f^{\prime}(x+)-f^{\prime}(x-) \bigr\vert \sqrt{\frac{C}{n} \biggl(1+\frac{1}{\rho}\biggr) (1+x)} \\& \qquad {}+\frac{C}{n}\biggl(1+\frac{1}{\rho}\biggr) \frac{(1+x)}{x^{2}} \bigl\vert f(2x)-f(x)-x f'(x+) \bigr\vert + \frac{x}{\sqrt{n}}\bigvee_{x}^{x+x/\sqrt {n}} \bigl(f'_{x}\bigr) \\& \qquad {}+\frac{C}{n}\biggl(1+\frac{1}{\rho}\biggr) \biggl(1+ \frac{1}{x}\biggr)\sum_{k=1}^{[\sqrt{n}]} \bigvee_{x}^{x+x/\sqrt{k}}f'_{x} +\frac{C}{n}\biggl(1+\frac{1}{\rho}\biggr) (1+x) \biggl( \frac{M+ \vert f(x) \vert }{x^{2}}+4M\biggr) \\& \qquad {}+ \bigl\vert f'(x+) \bigr\vert \sqrt{ \frac{C}{n}\biggl(1+\frac {1}{\rho}\biggr) (1+x)}, \end{aligned}$$

*where* $\bigvee_{a}^{b}f(x) $ *denotes the total variation of* $f(x) $ *on* $[a,b] $ *and* $f^{\prime}_{x}$ *is defined by*

39 $$\begin{aligned} f_{x}^{\prime}(t)= \textstyle\begin{cases} f^{\prime}(t)-f^{\prime}(x-), & 0\leq t< x, \\ 0, & t=x, \\ f^{\prime}(t)-f^{\prime}(x+) & x< t< \infty. \end{cases}\displaystyle \end{aligned}$$

### Proof

Since $\mathcal{L}_{n}^{\rho}(1;x)=1$, using (38), for every $x\in (0,1)$ we get

40 $$\begin{aligned} \mathcal{L}_{n}^{\rho}( f;x) -f(x) =& \int_{0}^{\infty}K_{n}^{\rho}(x,t) \bigl(f(t)-f(x)\bigr) \,dt \\ =& \int_{0}^{\infty}K_{n}^{\rho}(x,t) \int_{x}^{t}f^{\prime}(u) \,du \,dt. \end{aligned}$$

For any $f\in \operatorname{DBV}(R_{0}^{+})$, from (39) we may write

41 $$\begin{aligned} f^{\prime}(v) =&\bigl(f'\bigr)_{x}(v)+ \frac{1}{2}\bigl(f^{\prime}(x+)+f^{\prime}(x-)\bigr) + \frac{1}{2}\bigl( f^{\prime}(x+)-f^{\prime}(x-)\bigr) \operatorname{sgn}(v-x) \\ &{}+E _{x}(v)\biggl[ f^{\prime}(v)-\frac{1}{2}\bigl( f^{\prime}(x+)+f^{\prime}(x-)\bigr)\biggr], \end{aligned}$$

where

$$E _{x}(u)= \textstyle\begin{cases} 1,&v=x, \\ 0, &v\neq x. \end{cases} $$

We get

42 $$\begin{aligned} \int_{0}^{\infty}\biggl( \int_{x}^{t}\biggl(f^{\prime}(u)- \frac{1}{2}\bigl(f^{\prime}(x+)+f^{\prime}(x-)\bigr) \biggr)E_{x}(v)\,dv\biggr) K_{n}^{\rho}(x,t)\,dt=0. \end{aligned}$$

Using Lemma 2.2 and applying the Cauchy-Schwarz inequality, we obtain

43 $$ \begin{gathered}[b] \int_{0}^{\infty}\biggl( \int_{x}^{t}\frac{1}{2}\bigl( f^{\prime}(x+)+f^{\prime}(x-)\bigr) \,dv\biggr) K_{n}^{\rho}(x,t)\,dt \\ \quad =\frac{1}{2}\bigl(f^{\prime}(x+)+f^{\prime}(x-)\bigr) \int_{0}^{\infty}( t-x) K_{n}^{\rho}(x,t)\,dt \\ \quad =\frac{1}{2}\bigl( f^{\prime}(x+)+f^{\prime}(x-)\bigr) \mathcal{L}_{n}^{\rho}\bigl( (t-x);x\bigr) =\frac{1}{2} \bigl( f^{\prime}(x+)+f^{\prime}(x-)\bigr) \biggl(\frac {A_{t_{1}}(1,1)+A_{t_{2}}(1,1)}{n A(1,1)} \biggr) \end{gathered} $$

and

44 $$ \begin{gathered}[b] \int_{0}^{\infty}K_{n}^{\rho}(x,t) \biggl( \int_{x}^{t}\frac{1}{2}\bigl(f^{\prime}(x+)-f^{\prime}(x-) \bigr) \operatorname{sgn}(v-x)\,dv\biggr) \,dt \\ \quad \leq\frac{1}{2} \bigl\vert f^{\prime}(x+)-f^{\prime}(x-) \bigr\vert \bigl(\mathcal{L}_{n}^{\rho}\bigl(( t-x) ^{2};x\bigr)\bigr) ^{1/2} \\ \quad \leq\frac{1}{2} \bigl\vert f^{\prime}(x+)-f^{\prime}(x-) \bigr\vert \sqrt{\frac{C}{n}\biggl(1+\frac{1}{\rho}\biggr) (1+x)}. \end{gathered} $$

Using Lemma 2.2 and equations (40)-(44), we obtain

45 $$ \begin{aligned}[b] \bigl\vert \mathcal{L}_{n}^{\rho}(f;x)-f(x) \bigr\vert &\leq \frac {1}{2}\bigl( f^{\prime}(x+)+f^{\prime}(x-) \bigr) \biggl(\frac {A_{t_{1}}(1,1)+A_{t_{2}}(1,1)}{n A(1,1)}\biggr) \\ &\quad {}+\frac{1}{2} \bigl\vert f^{\prime}(x+)-f^{\prime}(x-) \bigr\vert \sqrt{\frac{C}{n}\biggl(1+\frac{1}{\rho}\biggr) (1+x)}+ \vert I_{1} \vert + \vert I_{2} \vert , \end{aligned} $$

where

$$\begin{aligned} I_{1}= \int_{0}^{x} \int_{x}^{t}\bigl(\bigl(f' \bigr)_{x}(v)\,dv\bigr) K_{n}^{\rho}(x,t)\,dt \end{aligned}$$

and

$$\begin{aligned} I_{2}= \int_{x}^{1} \int_{x}^{t}\bigl(\bigl(f' \bigr)_{x}(v)\,dv\bigr) K_{n}^{\rho}(x,t)\,dt. \end{aligned}$$

Since we know $\int_{a}^{b}d_{t}\xi_{n}^{\rho}(x,t)\leq1$, for all $[a,b]\subseteq R_{0}^{+}$, using integration by parts and applying Lemma 7.1 and substituting $y=x-x/\sqrt{n}$, we get

$$\begin{aligned} I_{1} =& \biggl\vert \int_{0}^{x} \int_{x}^{t}\bigl( \bigl(f' \bigr)_{x}(v)\,dv\bigr)\, d_{t}\xi _{n}^{\rho}(x,t) \biggr\vert \\ =& \biggl\vert \int_{0}^{x}\xi_{n}^{\rho}(x,t) \bigl(f'\bigr)_{x}(t)\,dt \biggr\vert \\ \leq& \int_{0}^{y} \bigl\vert \bigl(f' \bigr)_{x}(t) \bigr\vert \bigl\vert \xi _{n}^{\rho}(x,t) \bigr\vert \,dt+ \int_{y}^{x} \bigl\vert \bigl(f' \bigr)_{x}(t) \bigr\vert \bigl\vert \xi_{n}^{\rho}(x,t) \bigr\vert \,dt \\ \leq&\frac{C}{n}\biggl(1+\frac{1}{\rho}\biggr) (1+x) \int_{0}^{y}\bigvee_{t}^{x} \bigl(\bigl(f'\bigr)_{x}\bigr) (x-t)^{-2}\,dt+ \int_{y}^{x}\bigvee_{t}^{x} \bigl( \bigl(f'\bigr)_{x}\bigr) \,dt \\ \leq&\frac{C}{n}\biggl(1+\frac{1}{\rho}\biggr) (1+x) \int_{0}^{x-x/\sqrt{n}}\bigvee_{t}^{x} \bigl( \bigl(f^{\prime}\bigr)_{x}\bigr) ( x-t)^{-2}\,dt+ \frac{x}{\sqrt{n}}\bigvee_{x-x/\sqrt{n}}^{x}\bigl( \bigl(f'\bigr)_{x}\bigr). \end{aligned}$$

Substituting $v=x/(x-t)$, we get

$$\begin{aligned}& \frac{C}{n}\biggl(1+\frac{1}{\rho}\biggr) (1+x) \int_{0}^{x-x/\sqrt {n}}(x-t)^{-2}\bigvee _{t}^{x}\bigl(\bigl(f^{\prime}\bigr)_{x}\bigr) \,dt \\& \quad =\frac{C}{n}\biggl(1+\frac{1}{\rho}\biggr) (1+x)x^{-1} \int_{1}^{\sqrt {n}}\bigvee_{x-x/u}^{x} \bigl(\bigl(f^{\prime}\bigr)_{x}\bigr) \,dv \\& \quad \leq\frac{C}{n}\biggl(1+\frac{1}{\rho}\biggr) (1+x)x^{-1}\sum_{k=1}^{[\sqrt {n}]} \int_{k}^{k+1}\bigvee_{x-x/k}^{x} \bigl(\bigl(f^{\prime}\bigr)_{x}\bigr) \,dv \\& \quad \leq\frac{C}{n}\biggl(1+\frac{1}{\rho}\biggr) \biggl(1+ \frac{1}{x}\biggr)\sum_{k=1}^{[\sqrt{n}]} \bigvee_{x-x/k}^{x}\bigl( \bigl(f'\bigr)_{x}\bigr). \end{aligned}$$

Thus,

46 $$\begin{aligned} \vert I_{1} \vert \leq\frac{C}{n}\biggl(1+ \frac{1}{\rho}\biggr) \biggl(1+\frac {1}{x}\biggr)\sum _{k=1}^{[ \sqrt{n}]} \bigvee_{x-x/k}^{x} \bigl( \bigl(f^{\prime}\bigr)_{x}\bigr) +\frac{x}{\sqrt{n}} \bigvee_{x-x/\sqrt{n}}^{x}\bigl(\bigl(f^{\prime} \bigr)_{x}\bigr). \end{aligned}$$

Again, using integration by parts, applying the Cauchy-Schwarz inequality, Lemma 7.1 and substituting $z=x+(1-x)/\sqrt{n}$, we get

47 $$ \begin{aligned}[b] \vert I_{2} \vert &= \biggl\vert \int_{x}^{\infty} \int _{x}^{t}\bigl(\bigl(f' \bigr)_{x}(v)\,dv\bigr) K_{n}^{\rho}(x,t)\,dt \biggr\vert \\ &= \biggl\vert \int_{x}^{2x} \int_{x}^{t}\bigl(\bigl(f' \bigr)_{x}(v)\,dv\bigr) d_{t}\bigl(1-\xi _{n}^{\rho}(x,t) \bigr)+ \int_{2x}^{\infty} \int_{x}^{t}\bigl( \bigl(f' \bigr)_{x}(v)\,dv\bigr)K_{n}^{\rho}(x,t)\,dt \biggr\vert \\ &= \biggl\vert \biggl[ \int_{x}^{t}\bigl(\bigl(f' \bigr)_{x}(v)\,dv\bigr) \bigl(1-\xi_{n}^{\rho}(x,t) \bigr)\biggr]_{x}^{2x} \biggr\vert + \biggl\vert \int_{x}^{2x}\bigl(f' \bigr)_{x}(t) \bigl(1-\xi _{n}^{\rho}(x,t)\bigr)\,dt \biggr\vert \\ &\quad {}+ \biggl\vert \int_{2x}^{\infty} \int_{x}^{t}\bigl(\bigl(f'(v)-f'(x+) \bigr)\,dv\bigr) K_{n}^{\rho}(x,t) \,dt \biggr\vert \\ &\leq \biggl\vert \int_{x}^{2x}\bigl(\bigl(f' \bigr)_{x}(v)\,dv\bigr) \bigl(1-\xi_{n}^{\rho}(x,2x) \bigr)\biggr\vert + \biggl\vert \int_{x}^{2x}\bigl(f' \bigr)_{x}(t) \bigl(1-\xi_{n}^{\rho}(x,t)\bigr)\,dt \biggr\vert \\ &\quad {}+ \biggl\vert \int_{2x}^{\infty}f(t)K_{n}^{\rho}(x,t)\,dt \biggr\vert + \bigl\vert f(x) \bigr\vert \biggl\vert \int_{2x}^{\infty}K_{n}^{\rho}(x,t)\,dt \biggr\vert \\ &\quad {} + \bigl\vert f'(x+) \bigr\vert \biggl\vert \int_{2x}^{\infty}\bigl((t-x)\bigr)K_{n}^{\rho}(x,t)\,dt \biggr\vert \\ &\leq\frac{C}{n}\biggl(1+\frac{1}{\rho}\biggr)\frac{(1+x)}{x^{2}} \biggl\vert \int _{x}^{2x}(\bigl(f'(v)-f(x+) \bigr)\,dv \biggr\vert + \biggl\vert \int_{x}^{x+x/\sqrt {n}}f'_{x}(t)\,dt \biggr\vert \\ &\quad {}+\frac{C}{n}\biggl(1+\frac{1}{\rho}\biggr) (1+x) \biggl\vert \int^{2x}_{x+x/\sqrt {n}}(t-x)^{-2}f'_{x}(t)\,dt \biggr\vert + \biggl\vert \int_{2x}^{\infty}f(t)K_{n}^{\rho}(x,t)\,dt \biggr\vert \\ &\quad {}+ \bigl\vert f(x) \bigr\vert \biggl\vert \int_{2x}^{\infty}K_{n}^{\rho}(x,t)\,dt \biggr\vert + \bigl\vert f'(x+) \bigr\vert \biggl( \int_{2x}^{\infty}(t-x)^{2}K_{n}^{\rho}(x,t)\,dt \biggr)^{1/2}. \end{aligned} $$

By substituting $t=x+\frac{x}{u}$ and proceeding in a similar way to $I_{1}$, we get

48 $$\begin{aligned} \vert I_{2} \vert \leq&\frac{C}{n}\biggl(1+ \frac{1}{\rho}\biggr)\frac {(1+x)}{x^{2}} \bigl\vert f(2x)-f(x)-x f'(x+) \bigr\vert +\frac{x}{\sqrt {n}}\bigvee _{x}^{x+x/\sqrt{n}}\bigl(f'_{x}\bigr) \\ &{}+\frac{C}{n}\biggl(1+\frac{1}{\rho}\biggr) \biggl(1+ \frac{1}{x}\biggr)\sum_{k=1}^{[\sqrt {n}]} \bigvee_{x}^{x+x/\sqrt{k}}f'_{x}+ \int_{2x}^{\infty}M\bigl(1+t^{2} \bigr)K_{n}^{\rho}(x,t)\,dt \\ &{}+ \bigl\vert f(x) \bigr\vert \biggl\vert \int_{2x}^{\infty}K_{n}^{\rho}(x,t)\,dt \biggr\vert + \bigl\vert f'(x+) \bigr\vert \sqrt{ \frac {C}{n}\biggl(1+\frac{1}{\rho}\biggr) (1+x)}. \end{aligned}$$

Now for $t\geq2x$, we may write $t\leq2(t-x)$ and $x\leq t-x$. Now using Lemma 7.1, we obtain

49 $$\begin{aligned}& \int_{2x}^{\infty}M\bigl(1+t^{2} \bigr)K_{n}^{\rho}(x,t)\,dt+ \bigl\vert f(x) \bigr\vert \int_{2x}^{\infty}K_{n}^{\rho}(x,t)\,dt \\& \quad \leq\frac{M}{x^{2}} \int_{2x}^{\infty}(t-x)^{2}K_{n}^{\rho}(x,t)\,dt+4M \int_{2x}^{\infty}(t-x)^{2}K_{n}^{\rho}(x,t)\,dt \\& \begin{gathered}[b] \qquad {}+ \frac{ \vert f(x) \vert }{x^{2}} \int_{2x}^{\infty}(t-x)^{2}K_{n}^{\rho}(x,t)\,dt \\ \quad \leq\frac{C}{n}\biggl(1+\frac{1}{\rho}\biggr) (1+x) \biggl( \frac{M+ \vert f(x) \vert }{x^{2}}+4M\biggr). \end{gathered} \end{aligned}$$

Collecting the estimates (45)-(49), we get the required result. □

## Conclusion

A link between Szász-Durrmeyer type operators and multiple Appell polynomials has been established. The quantitative Voronovskaya type theorem and the Grüss-Voronovskaya type theorem have been proved. A local approximation result and the weighted approximation theorem have been discussed besides the approximation of functions whose derivatives are locally of bounded variation.
